# Long noncoding RNA LINC01410 promotes the tumorigenesis of neuroblastoma cells by sponging microRNA‐506‐3p and modulating WEE1

**DOI:** 10.1002/cam4.3398

**Published:** 2020-09-04

**Authors:** Jie Mi, Yang Han, Jin Zhang, Xiwei Hao, Maoqing Xing, Cong Shang

**Affiliations:** ^1^ Department of Pediatric Surgery The Affiliated Hospital of Qingdao University Qingdao City Shandong Province China; ^2^ Department of Pediatric Stomatological Stomatological Hospital of Qingdao City Qingdao City Shandong Province China; ^3^ Department of Respiratory Qingdao Women and Children's Hospital Qingdao City Shandong Province China

**Keywords:** long noncoding RNA LINC01410, microRNA‐506‐3p, neuroblastoma, proliferation, WEE1

## Abstract

**Objective:**

Neuroblastoma (NBL) is an extra‐cranial solid tumor in children. This study was attempted to investigate the regulatory mechanism of long noncoding RNA LINC01410 (LINC01410) on NBL.

**Methods:**

The expression of LINC01410, miR‐506‐3p, and WEE1 in NBL was evaluated by quantitative real time polymerase chain reaction. The proliferation and colony formation ability of NBL cells were analyzed by MTT and colony formation assay. Flow cytometry assay was executed to measure the apoptosis and cell cycle. Dual‐luciferase reporter assay was used to detect the targeted relationships among LINC01410, miR‐506‐3p, and WEE1. Additionally, the role of LINC01410 on NBL in vivo was evaluated according to a tumor xenograft model.

**Results:**

The expression of LINC01410 and WEE1 was enhanced and miR‐506‐3p was inhibited in NBL. LINC01410 knockdown attenuated the cell proliferation, colony formation ability, and inhibited tumor growth. Moreover, LINC01410 silencing facilitated the apoptosis and arrested the cell cycle. LINC01410 interacted with miR‐506‐3p to elevate the WEE1 expression in NBL. Additionally, miR‐506‐3p inhibition or WEE1 overexpression weakened the reduction effects of sh‐LINC01410 on cell proliferation, colony formation ability, apoptosis, and cell cycle.

**Conclusions:**

Knockdown of LINC01410 inhibited the development of NBL by miR‐506‐3p/WEE1 axis in vitro, which could serve as a potential therapeutic target for NBL therapy.

## Graphical abstract

LINC01410 was markedly increased in neuroblastoma (NBL). LINC01410 knockdown inhibited the tumorigenesis of NBL. MiR‐506‐3p was a target of LINC01410.

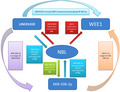

## 1 INTRODUCTION

As an extra‐cranial solid tumor,^1^ neuroblastoma (NBL) often occurs at early age and has high frequency of metastatic in children over 1 year old.^2^ Neuroblastoma has a heterogeneous presentation and prognosis, and the survival for high‐risk NBL is under 50%.^3^ Increasing schemes have been developed to treat high‐risk patients, whereas medium‐ or low‐risk patients have received decreased therapy.^4^ So far, understanding the role of molecular changes has allowed for accurate risk stratification to guide NBL therapy.^5^ Therefore, it is indispensable to explore the key molecules for NBL treatment.

Long noncoding RNAs (lncRNAs) participate in processes of different malignancies, such as glioma,^6^ meningioma,^7^ and NBL.^8^ Some lncRNAs act as onco‐lncRNA of NBL.^9^, ^10^ Long noncoding RNA 01410 (LINC01410), one of the lncRNAs, has been found to take part in a host of cancers. For instance, LINC01410 accelerates tumorigenesis of colon cancer through suppressing miR‐3128.^11^ LINC01410 regulates miR‐124‐3p to increase the SMAD5 expression, elevating cholangiocarcinoma cell proliferation, and migration rates.^12^ LINC01410 contributes to angiogenesis and metastasis in gastric cancer via inhibiting the miR‐532‐5p expression.^13^ However, the underlying mechanism of LINC01410 in NBL development remains unclear.

MicroRNAs (miRNAs) have been proved to inhibit translation through interacting with 3ʹ UTR.^14^ As biological molecules, miRNAs are involved in several cancers including NBL.^15^, ^16^ Some miRNAs have antitumor effects on biological processes of NBL. MiR‐137 serves as an anti‐oncogene in NBL via suppressing KDM1A.^17^ MiR‐335 inhibits the cell invasiveness of NBL by regulating noncanonical transforming growth factor‐beta (TGF‐β) pathway.^18^ Importantly, miR‐506 negatively mediates NBL cell proliferation and metastasis via targeting ROCK1.^19^ Despite these researches, the specific regulatory relationship between LINC01410 and miR‐506‐3p in NBL remains undefined.

WEE1, a tyrosine kinase, participates in the progression of cell cycle and DNA replication.^20^, ^21^ Additionally, WEE1 acts as a therapeutic target for tumor.^22^ WEE1 has been found in glioblastoma,^23^ osteosarcoma,^24^ and melanoma.^25^ It is noteworthy that miR‐497 induces NBL cell apoptosis through inhibiting the WEE1 expression.^26^ Nevertheless, the potential regulatory mechanism of LINC01410 related to the miR‐506‐3p/WEE1 axis in NBL is still unknown.

Herein, we evaluated the expression and functional role of LINC01410 in NBL. The regulatory relationship between LINC01410 and miR‐506‐3p was further investigated. Then, we explored whether WEE1 is a downstream target of miR‐506‐3p. An LINC01410/miR‐506‐3p/WEE1 regulatory axis in NBL pathogenesis was identified. Our study may uncover a new therapeutic target for NBL.

## 2 MATERIALS AND METHODS

### 2.1 Tissue samples

Total 61 children with NBL (28 males and 33 females) were collected between January 2017 and June 2018. None of the patients were performed preoperative systemic or local treatment. NBL tissues and adjacent noncancerous tissues were collected. Patient cohort was separated into high LINC01410 (n = 31) and low LINC01410 groups (n = 30) according to the median LINC01410 expression. This study was permitted by our hospital ethics committee, and informed consents were obtained from each patient or their guardian.

### 2.2 Cell culture

Human NBL cell lines (SK‐N‐SH, IMR‐32, Kelly, and SH‐SY5Y) and normal dorsal root ganglia (DG) cells were gained from the American Type Culture Collection. Cells were cultured at 37°C in dulbecco's minimum essential medium (DMEM, Invitrogen) with 10% fetal bovine serum (Invitrogen) with 5% CO_2_.

### 2.3 Cell transfection

A short hairpin RNA was designed to target regions of LINC01410, as well as its corresponding scramble negative control (NC) was synthesized by GenePharma. Then, they were inserted into the pGLVU6/Puro vector (GenePharma) to construct sh‐LINC01410 and sh‐NC. MiR‐506‐3p mimics, miR‐506‐3p inhibitor, miR‐NC, pcDNA3.1‐NC (pcDNA‐NC), and pcDNA‐WEE1 were synthesized by GenePharma. Kelly and IMR‐32 cells grown to 85% confluence were transfected or co‐transfected with these above agents using Lipofectamine 3000 reagent (Invitrogen). After 48 hours of transfection, cells were used for further assays.

### 2.4 Quantitative real time polymerase chain reaction and western blot

Quantitative real time polymerase chain reaction (qRT‐PCR) and western blot were performed as described previously.^27^ The primers were listed in Table 1. GAPDH, U6, and β‐actin were used for the normalization of LINC01410, miR‐506‐3p, and WEE1, respectively. The antibodies for western blot analysis were: anti‐WEE1 primary antibody (1:1000, WH0007465M1MSDS; Sigma), anti‐β‐Actin (1:5000, SAB2701711MSDS; Sigma), and horseradish peroxidase (HRP)‐conjugate secondary antibody (1:5000, 12‐349MSDS; Sigma). The protein bands were visualized by enhanced chemiluminescence exposure solution, and quantified by ImageLab software (Bio‐Rad).

**TABLE 1:** Primers sequences

Name of primer	Sequences (5ʹ‐3ʹ)
LINC01410‐F	GTGACAAGAATGGCCCAAGC
LINC01410‐R	ACTGTGCACCTGTTACACCA
GAPDH‐F	CGCTCTCTGCTCCTCCTGTTC
GAPDH‐R	ATCCGTTGACTCCGACCTTCAC
miR‐506‐3p ‐F	TAAGGCACCCTTCTGAGTAGA
miR‐506‐3p ‐R	GCGAGCACAGAATTAATACGAC
U6‐F	AACGAGACGACGACAGAC
U6‐R	GCAAATTCGTGAAGCGTTCCATA
WEE1‐F	GGCTCTGTTGATGAGCAGAACGCTT
WEE1‐R	CTCAAGCCTCGGCGGCCAACTTGC
β‐actin‐F	ATTGCCGACAGGATGCAGAA
β‐actin‐R	CAAGATCATTGCTCCTCCTGAGCGCA

### 2.5 Mouse xenograft tumor model

Twelve male BALB/c nude mice (4 week old) were purchased from Beijing HFK Bioscience Co, Ltd. Mice were fed in a standalone environment, at 22‐25°C and 55%‐66% humidity, with a normal diet. Mice were randomly divided into sh‐NC group (n = 6) and sh‐LINC01410 group (n = 6). Next, sh‐NC or sh‐LINC01410 stably transfected Kelly cells (1 × 10^6^ cells) were subcutaneously inoculated into the dorsal back region of mice as described earlier.^28^ Tumor volume was measured 1 week after inoculation, and calculated as follows: 0.5 × length × width^2^. After the last measurement at the fourth week postinjection, mice were sacrificed by cardiac puncture, and tumor weight was tested. All animal experiments and programs were approved by the Animal Care and Use Committee of our hospital.

### 2.6 3‐(4,5‐Dimethylthiazol‐2‐yl)‐2,5‐diphenyltetrazolium bromide (MTT) assay

The Kelly and IMR‐32 cells with different transfection were seeded into 96‐well plates (4 × 10^3^ cells/well) and cultured at 37°C with 5% CO_2_. Cell proliferation was assessed using the MTT cell proliferation assay kit (Sigma).

### 2.7 Colony formation assay

The Kelly and IMR‐32 cells after transfection were digested with trypsin and seeded into six‐well plates (4 × 10^3^ cells/well) for 2 weeks at 37°C. Subsequently, cells were fixed with methanol and stained with 0.1% crystal violet. After washing with distilled water, the colonies were investigated via an inverted microscope.

### 2.8 Flow cytometry assay

The cell cycle and cell apoptosis were analyzed by flow cytometer (Attune NxT; Thermo Fisher Scientific) as described previously.^29^

### 2.9 Dual‐luciferase reporter assay

The potential binding sites of LINC01410 and miR‐506‐3p or miR‐506‐3p and WEE1 were predicted by Starbase or TargetScan. The sequence of LINC01410 including the binding site of miR‐506‐3p was cloned into psiCHECK‐2 vectors (Promega) containing luciferase gene to construct LINC01410‐Wt, LINC01410‐Mut, WEE1‐Wt, and WEE1‐Mut. Then, they were co‐transfected with miR‐NC and miR‐506‐3p mimics into Kelly and IMR‐32 cells with Lipofectamine 3000 (Invitrogen) for 48 hours. Luciferase activity was examined through dual‐luciferase assay system (Promega).

### 2.10 Statistical analysis

Statistical data were performed on GraphPad Prism 7.0 (GraphPad Software, Inc) and were displayed as mean ± SD. The differences between two groups or among multiple groups were assessed by Student's *t* test or one‐way ANOVA followed by Tukey's post hoc test. The correlation significance was determined by Pearson correlation analysis. A *P* < .05 was considered statistically significant.

## 3 RESULTS

### 3.1 LINC01410 was markedly increased in NBL

**FIGURE 1:**
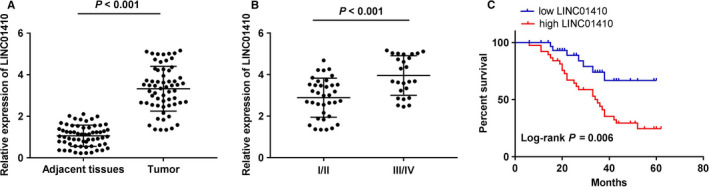
LINC01410 was markedly increased in neuroblastoma (NBL) patients. A, LINC01410 expression in tissues of NBL patients was examined by quantitative real time polymerase chain reaction (qRT‐PCR). *P* < .001 vs adjacent tissues; (B) LINC01410 expression in NBL patients at the TNM I/II and TNM III/IV was obtained by qRT‐PCR *P* < .001 vs I/II; (C) the relationship between LINC01410 and survival percent of NBL patients was measured through Kaplan‐Meier curves (long‐rank test: *P* = .006)

We first measured LINC01410 expression in tissues of NBL patients by qRT‐PCR. The LINC01410 expression in tumor tissues was markedly increased compared with the adjacent tissues in NBL patients (*P* < .001; Figure 1A). Additionally, qRT‐PCR showed that the LINC01410 expression in tissues from patients in TNM III/IV was higher than that in TNM I/II (*P* < .001; Figure 1B). NBL patients with lower LINC01410 expression exhibited a higher survival percent (*P* = .006; Figure 1C). The clinicopathological features displayed that the LINC01410 expression had significant correlation with World Health Organization (WHO) grade in NBL patients (*P* < .05, Table 2).

**TABLE 2:** Correlation between LINC01410 expression and clinicopathological features in children with neuroblastoma

Characteristics	n	LINC01410 (Low) 30	(High) 31	*P* value
Age				.237
<1 y	20	12	8	
≥1 y	41	18	23	
Gender				.363
Male	28	12	16	
Females	33	18	15	
Diameter				.886
<3 cm	34	17	17	
≥3 cm	27	13	14	
Metastasis				.086
No	36	21	15	
Yes	25	9	16	
WHO grade				.025*
Ⅰ + Ⅱ	36	22	14	
Ⅲ + Ⅳ	25	8	17	

Abbreviation: WHO, World Health Organization.

* *P* < .05.

### 3.2 Silencing of LINC01410 inhibited tumorigenesis of NBL

**FIGURE 2:**
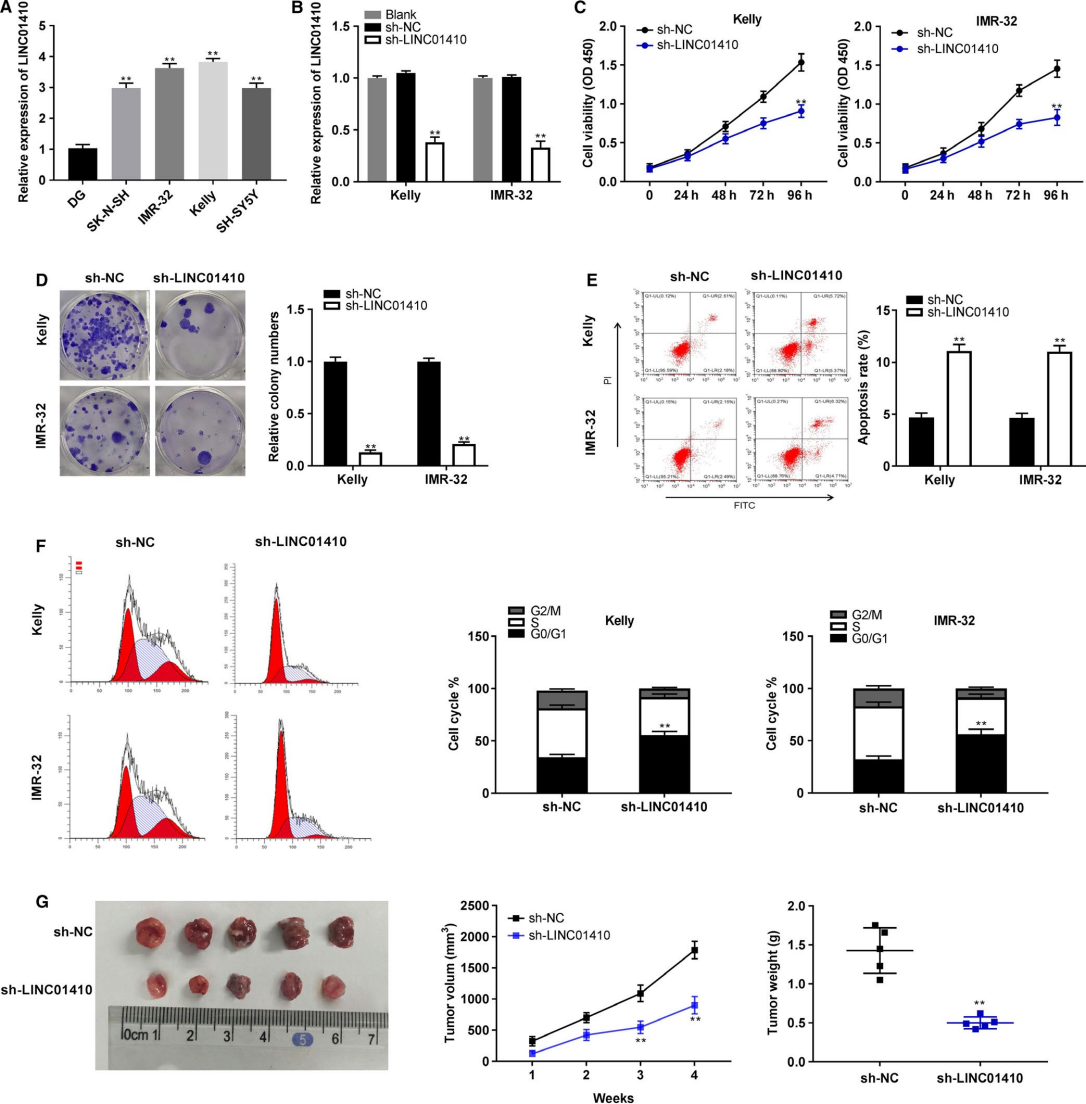
Silencing of LINC01410 inhibited tumorigenesis of neuroblastoma (NBL). A, Quantitative real time polymerase chain reaction was performed to text LINC01410 expression of in SK‐N‐SH, IMR‐32, Kelly, SH‐SY5Y, and DG cells. **P < .01 vs DG; (B) the transfection efficiency of sh‐negative control (NC) and sh‐LINC01410 in cells; (C) cell proliferation was detected by MTT assay; (D) the relative colony numbers of cells was confirmed through colony formation assay; (E, F) the apoptosis rate and cell cycle of cells were determined by flow cytometry; (G) tumor volume and tumor weight. **P < .01 vs sh‐NC

Quantitative real time polymerase chain reaction displayed that the LINC01410 expression was considerably increased in SK‐N‐SH, IMR‐32, Kelly, and SH‐SY5Y cells (*P* < .01; Figure 2A). The Kelly and IMR‐32 cells were used for subsequent assays on account of relative high expression of LINC01410. As shown in Figure 2B, the knockout efficiency of sh‐LINC01410 was detected. Sh‐LINC01410 could successfully silence the LINC01410 expression in Kelly and IMR‐32 cells (*P* < .01). MTT assay discovered that sh‐LINC01410 could dramatically decline the proliferation of Kelly and IMR‐32 cells at 72 and 96 hours post‐culturing (*P* < .01; Figure 2C). Silencing of LINC01410 visibly reduced the relative colony numbers of Kelly and IMR‐32 cells (*P* < .05; Figure 2D). Furthermore, we investigated the functions of LINC01410 silencing on modulating apoptosis and cell cycle via flow cytometry. LINC01410 knockdown could elevate the apoptosis rate of Kelly and IMR‐32 cells (*P* < .01; Figure 2E). Silencing of LINC01410 not only caused an obvious accumulation in G0/G1 phase cells, but also led to a reduction of cells in S and G2/M phases (*P* < .01; Figure 2F). In vivo, LINC01410 knockdown could markedly reduce the tumor volume at 3 and 4 weeks after inoculation (*P* < .01), as well as reduce the tumor weight (*P* < .01; Figure 2G).

### 3.3 MiR‐506‐3p served as a target of LINC01410

**FIGURE 3:**
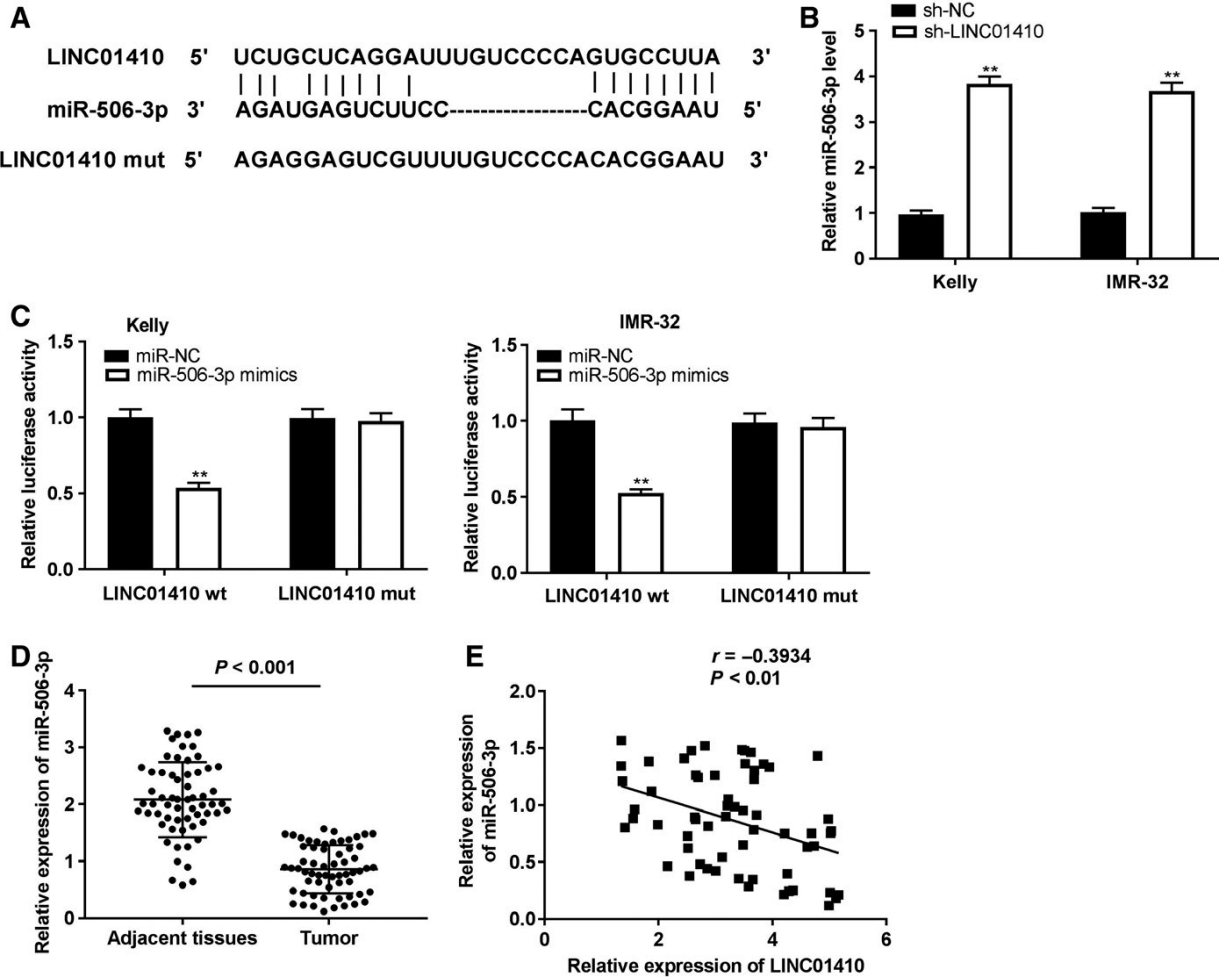
MiR‐506‐3p served as a target of LINC01410. A, Starbase exhibited the predicted binding site between LINC01410 and miR‐506‐3p; (B) MiR‐506‐3p expression in cells was detected by quantitative real time polymerase chain reaction (qRT‐PCR). **P < .01 vs sh‐negative control (NC); (C) relative luciferase activity in Kelly and IMR‐32 cells was assessed by dual‐luciferase reporter assay. **P < .01 vs miR‐NC; (D) MiR‐506‐3p expression in tissues of NBL patients was detected by qRT‐PCR. E, LINC01410 expression was negatively correlated with miR‐506‐3p

As depicted in Figure 3A, using starbase on‐line software, we found that LINC01410 contained the target site of miR‐506‐3p (Figure 3A). LINC01410 silencing could significantly increase the miR‐506‐3p expression in Kelly and IMR‐32 cells (*P* < .01; Figure 3B). Then, miR‐506‐3p mimics reintroduction obviously attenuated the luciferase activity in Kelly and IMR‐32 cells transfected with LINC01410 Wt (*P* < .01; Figure 3C). Quantitative real time polymerase chain reaction exhibited that the miR‐506‐3p expression was obviously suppressed in tumor tissues of NBL patients (*P* < .001; Figure 3D). A negative correlation between the LINC01410 and miR‐506‐3p expression was displayed in NBL tissues (N = 61, *r* = −.3934, *P* < .01; Figure 3E).

### 3.4 MiR‐506‐3p inhibited tumorigenesis of NBL cells

**FIGURE 4:**
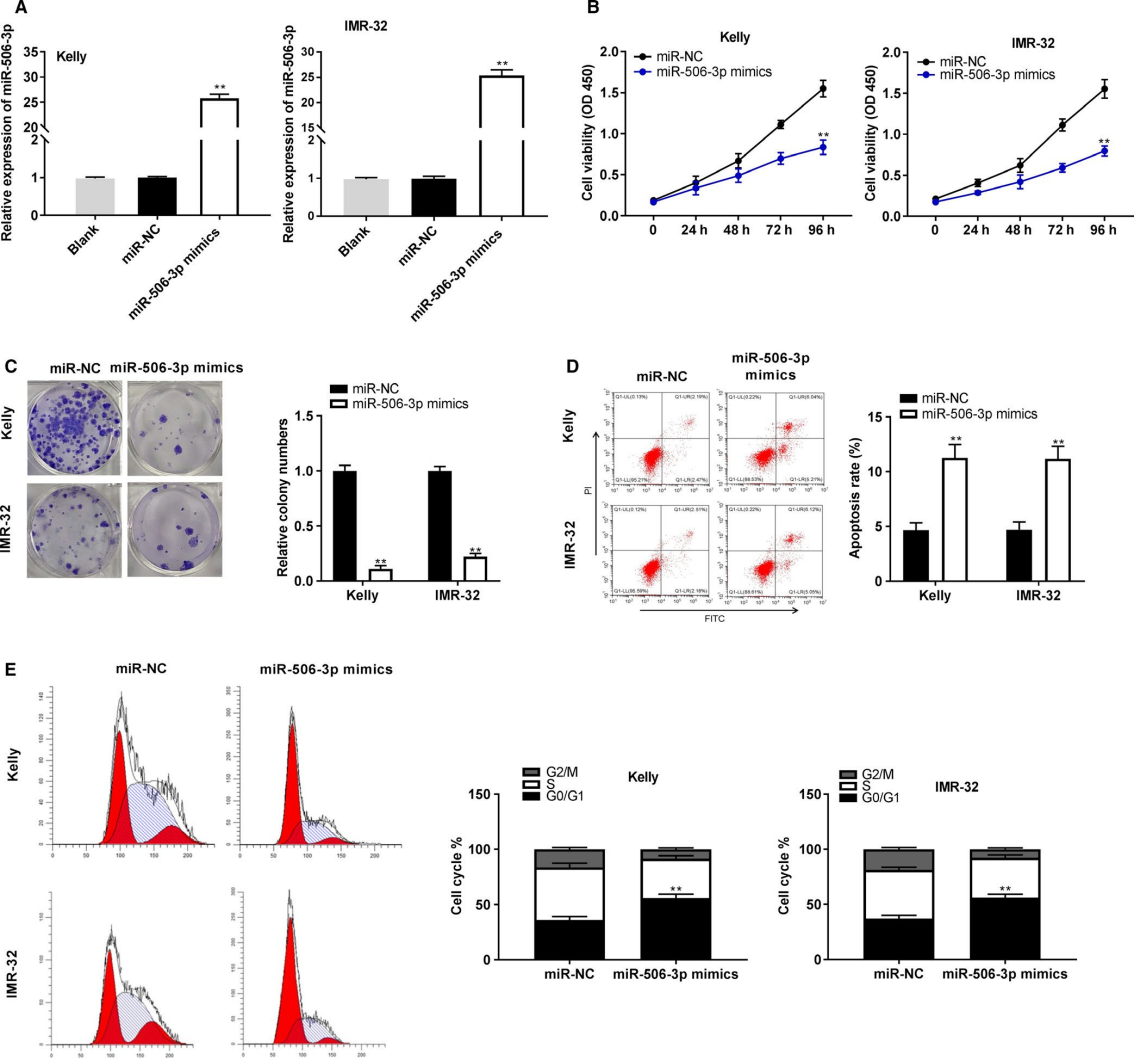
MiR‐506‐3p attenuated tumorigenesis of neuroblastoma (NBL) cells. A, The transfection efficiency of miR‐negative control (NC) and miR‐506‐3p in cells; (B, C) MiR‐506‐3p overexpression suppressed cell proliferation and colony formation ability; (D, E) flow cytometry was executed to determine cell apoptosis and cell cycle. **P < .01 vs miR‐NC

To evaluate the biological functions of miR‐506‐3p in NBL, miR‐506‐3p was enhanced by the transfection of miR‐506‐3p mimics into Kelly and IMR‐32 cells (*P* < .01; Figure 4A). MiR‐506‐3p considerably decreased the proliferation and colony numbers of Kelly and IMR‐32 cells (*P* < .01; Figure 4B,C). Moreover, the apoptosis rate of Kelly and IMR‐32 cells was obviously elevated by miR‐506‐3p overexpression (*P* < .01; Figure 4D). Following the miR‐506‐3p enhanced, the proportion of cells in G0/G1 phase was markedly elevated, while reduced in S and G2/M phases (*P* < .01; Figure 4E).

### 3.5 WEE1 was targeted by miR‐506‐3p

**FIGURE 5:**
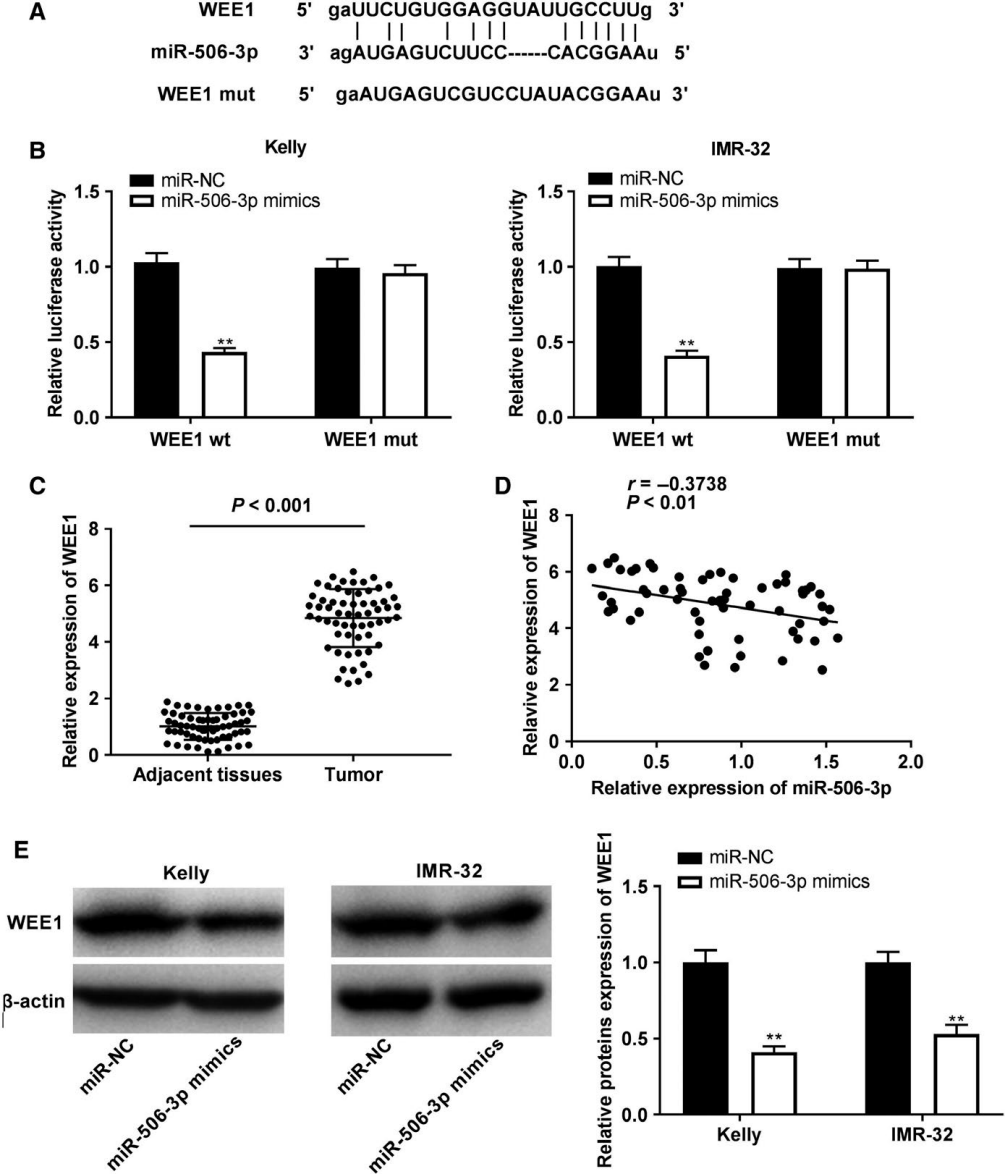
WEE1 was directly targeted by miR‐506‐3p. A, TargetScan displayed the predicted binding site between miR‐506‐3p and WEE1; (B) relative luciferase activity in Kelly and IMR‐32 cells was measured by dual‐luciferase reporter assay. **P < .01 vs miR‐negative control (NC); (C) WEE1 expression in tissues of neuroblastoma patients was detected by quantitative real time polymerase chain reaction. *P* < .001 vs adjacent tissues; (D) MiR‐506‐3p expression was negatively correlated with WEE1; (F) WEE1 protein expression i was assessed by western blot in cells. **P < .01 vs miR‐NC

TargetScan was used to predict the binding site for miR‐506‐3p on the 3ʹ UTR of WEE1 (Figure 5A). MiR‐506‐3p mimics obviously declined the luciferase activity of WT WEE1 reporter vector in Kelly and IMR‐32 cells (*P* < .01; Figure 5B). Additionally, qRT‐PCR revealed that WEE1 expression was markedly enhanced in tumor tissues of NBL patients (*P* < .001; Figure 5C). A negative correlation between miR‐506‐3p and WEE1 expression was discovered in NBL tissues (N = 61, *r* = −.3738, *P* < .01; Figure 5D). Interestingly, western blot uncovered that transfection of miR‐506‐3p mimics could inhibit the WEE1 protein expression (*P* < .01; Figure 5E).

### 3.6 Sh‐LINC01410 inhibited the tumorigenesis of NBL cells by targeting miR‐506‐3p/WEE1 axis

**FIGURE 6:**
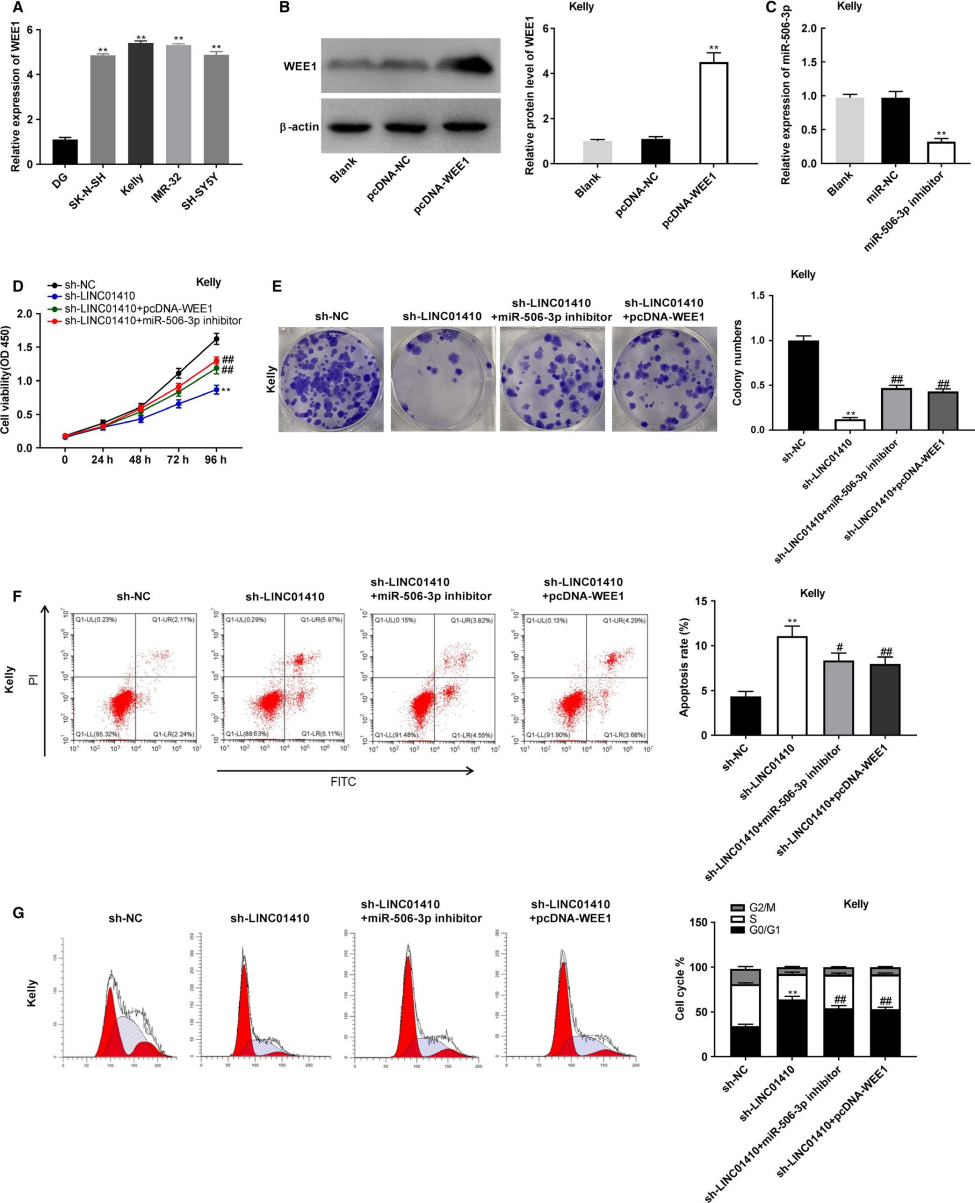
LINC01410 silencing attenuated tumorigenesis of neuroblastoma (NBL) cells by targeting miR‐506‐3p/WEE1 axis. A, WEE1 expression in SK‐N‐SH, IMR‐32, Kelly, SH‐SY5Y, and DG cells was examined by quantitative real time polymerase chain reaction. **P < .01 vs DG; (B) the protein expression of WEE1 in Kelly cells was evaluated by western blot. **P < .01 vs pcDNA‐negative control (NC); (C) the transfection efficiency of miR‐NC and miR‐506‐3p inhibitor in kelly cells. **P < .01 vs miR‐NC; (D) the proliferation of Kelly cells was assessed by MTT assay; (E) the relative colony numbers of Kelly cells was confirmed by colony formation assay; (F, G) the apoptosis rate and cell cycle of Kelly cells were determined by flow cytometry. **P < .01 vs sh‐NC; ^#^ *P* < .05, ^##^ *P* < .01 vs sh‐LINC01410

Quantitative real time polymerase chain reaction uncovered that the WEE1 expression was markedly upregulated in SK‐N‐SH, IMR‐32, Kelly, and SH‐SY5Y cells (*P* < .01; Figure 6A). The Kelly cells were used for subsequent assays. Western blot displayed that the WEE1 protein expression was increased by transfection of pcDNA‐WEE1 (*P* < .01; Figure 6B). As depicted in Figure 6C, miR‐506‐3p inhibition could markedly elevate the miR‐506‐3p expression in Kelly cells (*P* < .01). The proliferation and colony formation of Kelly cells were markedly inhibited by LINC01410 knockdown. Inhibition of miR‐506‐3p or overexpression of WEE1 markedly reversed the effects of sh‐LINC01410 exerted in Kelly cells (*P* < .01; Figure 6D,E). In addition, LINC01410 knockdown promoted the apoptosis rate and attenuated the cell cycle of Kelly cells (*P* < .01; Figure 6F,G). MiR‐506‐3p inhibition or WEE1 overexpression obviously weakened the promoting effect of sh‐LINC01410 on apoptosis rate (*P* < .05), as well as markedly weakened the suppression effect of sh‐LINC01410 on the cell cycle of Kelly cells (*P* < .01; Figure 6F,G).

## 4 DISCUSSION

Dysregulation of lncRNAs in multiple cancers including NBL is involved in the regulation of oncological behaviors.^30^, ^31^, ^32^ Here, the LINC01410 expression was markedly enhanced in NBL tissues and cells. Notably, LINC01410 is high‐expressed in diverse tumors, including gastric cancer,^13^ cervical cancer,^33^ and cholangiocarcinoma.^12^ In this study, high LINC01410 expression was positively associated with WHO grade in NBL patients, and conferred a survival disadvantage to NBL patients. Previous researches have illustrated that certain lncRNAs are upregulated and related to poor prognosis in NBL. For instances, LncRNA SNHG7 expression is evidently related to metastasis, INSS stage, and NBL patients with high lncRNA SNHG7 levels have poor outcomes.^29^ LncRNA *pancEts*‐1 elevates in NBL tissues, and is correlated with bad prognosis of NBL patients.^34^ Above all, our findings underline that LINC01410 may serve as a potential biomarker in NBL.

Long noncoding RNAs are related to the regulation of biological processes including carcinogenesis.^35^ LINC01410 has been proved to exhibit carcinogenic function in cancers. For instances, LINC01410 silencing retards cell proliferation and facilitates apoptosis via regulating miR‐3619‐5p/FOXM1 axis in thyroid carcinoma.^36^ Knockdown of LINC01410 attenuates cell growth and arrests cell cycle at G0/G1 phase via suppressing miR‐3128 in colon cancer.^11^ LINC01410 serves as an oncogenic that accelerates cholangiocarcinoma tumorigenesis by modulating the expression of SMAD5 via directly targeting miR‐124‐3p.^12^ In this study, LINC01410 knockdown restrained cell proliferation and colony formation ability, and arrested cell cycle, and accelerates cell apoptosis, suggesting that LINC01410 plays pivotal roles in the modulation of NBL progression in vitro. In addition, some lncRNAs serve as tumor promoters in NBL, and lncRNAs depletion lead to the inhibition of tumor growth in vivo. Long noncoding RNA RNA component of mitochondrial RNA processing endoribonuclease (RMRP) silencing retards NBL xenograft growth via regulating miR‐206/TACR1 axis.^37^ Inhibition of lncRNA XIST reduces the NBL tumor volume in mice.^38^ Long noncoding RNA DLX6‐AS1 silencing impedes NBL tumor growth.^39^ Similarly, LINC01410 knockdown alleviated the tumor growth in mice, indicating the important function of silencing LINC01410 in suppressing NBL tumorigenesis in vivo. Taken together, LINC01410 may be a promising therapeutic target for NBL.

Prior researches have showed that lncRNAs can target miRNAs to perform its oncogenic effects in NBL. For example, silencing of lncRNA SNHG7 hampers cell growth and promotes cell apoptosis of NBL by inhibiting the miR‐653‐5p expression.^29^ Long noncoding RNA RMRP functions as an oncogene in NBL via decreasing miR‐4518 expression.^37^ In this study, miR‐506‐3p was a target of LINC01410, suggesting that LINC01410 may mediate NBL by similar mechanisms above. Moreover, miR‐506‐3p is low‐expressed and acts as anti‐onco‐miR in diverse cancers. Previous studies have pointed out that miR‐506‐3p inhibits cell growth of pancreatic cancer and induces cell apoptosis,^40^ and attenuates tumorigenesis of osteosarcoma cell by suppressing RAB3D expression.^41^ Notably, miR‐506 hinders cell proliferation and metastasis of NBL via downregulating ROCK1.^19^ Here, the miR‐506‐3p expression was repressed and negatively related to LINC01410 expression. MiR‐506‐3p attenuated NBL cell proliferation, arrested cell cycle, and accelerated the apoptosis, and miR‐506‐3p inhibition markedly weakened the effects of LINC01410 silencing on NBL cells. Thus, LINC01410 may promote NBL progression via decreasing miR‐506‐3p expression in vitro.

Mounting data proved that miRNAs involves in cancers through mediating expression of oncogenes or tumor suppressors.^42^, ^43^ WEE1 is increased in different malignancies, and WEE1 knockdown exerts antitumor effects in numerous tumors.^44^, ^45^ Garimella et al have proved that WEE1 knockdown decreases the cell viability and promotes the cell cycle arrest in breast cancer.^46^ Russell et al have pointed out that the growth of NBL cells rely on WEE1 activity, and that knockdown of WEE1 acts as a therapeutic for NBL.^47^ Here, the WEE1expression was increased in NBL. Additionally, WEE1 can be involved in NBL progression as a target gene for miRNAs. MiR‐497 decreases cell viability and induces apoptosis of NBL through targeting WEE1.^26^ Our result substantiated that WEE1 was a target of miR‐506‐3p, and upregulation of WEE1 reverses the antitumor effects of sh‐LINC01410 on NBL cells. To sum up, LINC01410 increases the WEE1 expression by sponging miR‐506‐3p to exert antitumor effects in NBL cells.

## 5 CONCLUSIONS

In conclusion, LINC01410 expression was enhanced in NBL tissues and cells. LINC01410 served as an oncogenic lncRNA that accelerated NBL tumorigenesis by regulating the miR‐506‐3p/WEE1 axis in vitro. Thus, LINC01410 may be a potential therapeutic target for NBL.

## CONFLICT OF INTEREST

The authors declare that they have no conflicts of interest to disclose.

## AUTHOR CONTRIBUTION

Conceptualization: Jie Mi and Yang Han Formal analysis: Jie Mi, Yang Han, and Jin Zhang Investigation: Xiwei Hao and Maoqing Xing Methodology: Jie Mi and Cong Shang Writing—original draft: Jie Mi, Yang Han, and Jin Zhang Writing—review & editing: Xiwei Hao, Maoqing Xing, and Cong Shang Approval of final manuscript: all authors.

## Data Availability

All data are available through the corresponding author.
